# Development of ZnO Nanoflake Type Structures Using Silk Fibres as Template for Water Pollutants Remediation

**DOI:** 10.3390/polym12051151

**Published:** 2020-05-18

**Authors:** K. Jagajjanani Rao, Tarangini Korumilli, Akshaykumar KP, Stanisław Wacławek, Miroslav Černík, Vinod V. T. Padil

**Affiliations:** 1 Department of Biotechnology, Vel Tech Rangarajan Dr.Sagunthala R&D Institute of Science and Technology, Chennai, Tamil Nadu 600062, India; drktarangini@veltech.edu.in; 2 Tata Institute of Fundamental Research Hyderabad, Hyderabad Sy. No 36/P, Serilingampally Mandal, Hyderabad, Telangana 500107, India; kpakshay95@gmail.com; 3 Department of Nanomaterials in Natural Sciences, Institute for Nanomaterials, Advanced Technologies and Innovation (CXI), Technical University of Liberec (TUL), Studentská 1402/2, 1 461 17 Liberec, Czech Republic; stanislaw.waclawek@tul.cz

**Keywords:** silk fibers, ZnO, nanoflakes, photo-degradation, dye removal, antibacterial efficiency

## Abstract

We have fabricated ZnO nanoflake structures using degummed silk fibers as templates, via soaking and calcining the silk fibers bearing ZnO nanoparticles at 150 °C for 6 h. The obtained ZnO nanostructures were characterized using scanning electron microscopy (SEM), X-ray diffraction analysis (XRD), and UV-vis and fluorescence spectroscopic analysis. The size (~500–700 nm) in length and thicknesses (~60 nm) of ZnO nanoflakes were produced. The catalysis performances of ZnO nanoflakes on silk fibers (ZnSk) via photo-degradation of naphthalene (93% in 256 min), as well as Rose Bengal dye removal (~1.7 mM g^−1^) through adsorption from aqueous solution, were practically observed. Further, ZnSk displayed superb antibacterial activity against the tested model gram-negative *Escherichia coli* bacterium. The produced ZnSk has huge scope to be used for real-world water contaminants remediation applications.

## 1. Introduction

ZnO nanoparticles have significant technological applications, as they are well known for their catalytic, electrical, optoelectronic, antimicrobial properties, etc. [1]. They possess a large direct band gap of ~3.37 eV, and are used as functional devices in solar cells, and photocatalysts in remedial applications and sensor materials [2]. ZnO has vast areas of application, and a variety of nanostructures including nanoparticles, nanowires, nanorods, nanotubes, nanobelts, and other complex morphologies have been fabricated and reported [3]. Researchers suggest that nanostructures can be attached to host polymer materials such as porous resins, cellulose and silica, to minimize a hazard to humans and the environment derived from the uncontrolled release [4]. The nanoparticles fixed to the host materials make them bulkier and they can be easily removed and recovered after application, which makes them of potential use in removal of pollutants from the environment, e.g., to break down organochlorine pesticides, halogenated herbicides and azo dyes [5]. 

Taking a practical science point of view into consideration, recent research has been accelerated towards the development of novel composite materials using thermostable biopolymers (chitin, spongin, silk) and selected inorganic phases [6,7]. Using hydrothermal synthesis conditions primarily, the synergized concept uses extreme biomineralization and bioinspired materials chemistry for the generation of advanced and functional composite materials [6]. The name given to this approach and filed is “extreme biomimetics”, which produces materials with hierarchical and nanostructural organizations, alongside three-dimensional (3D) architecture at micro- and macrolevels [8,9]. Reported studies show biological templates with 3D morphology have been used as a matrix to generate TiO_2_, Zirconia, ZnO, hematite, germanium oxide composites, etc. [6,9,10]. For instance, spongin, a protein scaffold from marine demosponge, *Hippospongia communis* was used to produce TiO_2_ 3D composite structures using hydrothermal conditions (120 °C for 3 h) [6]. Likewise, lyophilized skeletons of marine sponges *Aplysina cauliformis* were used to produce novel chitin–GeO_2_ photoluminescent nanocomposites, using a hydrothermal reaction with a temperature of 185 °C [9]. In another study, β-chitinous scaffolds isolated from marine cephalopod *Sepia officinalis* to synthesize a template for the in vitro formation of ZnO nanostructure composites at 70 °C, which are extreme for the used biological material [10]. The key step in the above mentioned studies is thermal stability of the biopolymers during hydrothermal synthesis and processing [6,9,10]. Among inorganic nanomaterials, ZnO structures and composites are unique with antibacterial activity, wide bandgap, photocatalytic properties, and are known for having numerous practical applications [8,10].

Template directed synthesis of ZnO materials is gaining significant interest in research communities, and various porous and non-porous materials of ZnO have been synthesized [2,5]. Polyethylene glycol, copolymer gels, polyvinylpyrrolidone, nanofibres, etc., templates have been used to produce porous materials, while various soft biological materials, like butterfly wings, bacteria, bamboo, cotton, wool, silk fibers, etc., have been used to produce ZnO networks and hybrid materials [2,11,12]. The selection of natural and renewable sources to synthesize nanomaterials is imperative to sustainable development, and can limit hazardous wastes. Materials from biological sources with distinctive structures are plentiful in nature and are inexpensive and environmentally friendly. Exploring these structures for inorganic-organic nanocomposites, in particular with ZnO composites, holds significant challenges that need to be addressed. Process simplicity, more than one preparation step, pre- and post-treatments, high-temperature dependency, and a selection of suitable templates, etc., are key components, which affect the process pathway from the laboratory to commercial application.

Natural fibers from silkworms are well known for their biomedical applications, and recent studies display their potential as templates for nanoparticle synthesis [13,14] Degummed silk fibers are rich in glycine, alanine, and serine functional groups, which interact with metal ion containing precursors and can assist in nanoparticle deposition [15]. The application of silk materials for water treatment is a new area of research still in its infancy. Challenges like functionalization and impregnation with nanomaterials for applications like toxic pollutants removal, oil/solvent-water separation, smart textiles etc., are the key thrust areas in this field [15,16] Silk materials are biocompatible, biodegradable, and mechanically strong materials, readily available and suitable for water treatment applications, as they pose no disposal problem in the environment after their subsequent exhaustive use [15]. There are limited studies where research groups used silk materials as a template, and as an assistive substrate for the synthesis and growth of ZnO particles [11,12,17]. These involve multistep procedures and the use of harmful chemicals like N, N-dimethyl formamide (DMF). ZnO nanocomposites for toxic dyes removal, contaminant remediation, and water treatment are of scientific interest, and the use of silk fibers for the production of ZnO composites with simple preparation steps is a challenging task.

Our work focused on introducing a facile template assisted synthesis strategy to attain flake type ZnO nanoparticles. In this study, a green template source (silk fibers) was utilized with zinc acetate precursor, with a reasonably low temperature annealing process, to attain ZnO nanoflakes on silk fibers (ZnSk). The synthesized ZnSk were used for pollutant remediation studies, such as naphthalene degradation and Ross Bengal (RB) dye removal from the aqueous system successfully. The stated chemicals were selected as model pollutants, as they have profound effects on the environment. For instance, the photo-degradation of naphthalene is slow, with photochemical half-life usually beyond 100 h [18], and RB, a Xanthan dye extensively used in printing industries, insecticides, etc., produces the residual dye with the potential to pollute water [19]. Furthermore, the observed leach out of ZnO from the ZnSk is low; consequently, the synthesized ZnSk structures with decent antimicrobial activity against the tested *E. coli* bacteria may be useful for large-scale wastewater treatment applications in real-time settings.

## 2. Materials and Methods

### 2.1. Degumming Step

Dried cocoons of *Bombyxmori* silkworm were gifted by Silkworm Culture and Reeling Unit, Chebrolu, Andhra Pradesh, India. Further degumming steps were adopted from a reported study [20]. In brief, the obtained cocoons without insects were peeled into pieces and treated 3 times with 0.5 wt % aqueous Na_2_CO_3_ solution at 100 °C for 30 min. The process was repeated until the silk fibers tuned to white from pale yellow and the degumming solution stayed clear. Next, the samples obtained were washed with deionized water and naturally dried to get degummed silks.

### 2.2. Synthesis of ZnO Nanoflakes on Silk Fibres (ZnSk)

ZnO nanostructures were obtained on silk fibers by modifying the approach presented in a reported study using zinc acetate as a starting material [21]. In a typical experiment, 30 mM Zn(CH_3_COO)_2_·2H_2_O solution was prepared using a mixture of H_2_O: methanol: ethanol in the volumetric ratio of 5:4:6. A small amount of acetic acid was added to adjust the pH value to ~4.8 to prevent the formation of hydroxides. Then, the solution was homogeneously mixed using a sonicator and 1 mL of it added to degummed silk fibroin of 56.8 mg. The wetted silk fiber was transferred to a glass crucible and kept in an oven at 150 °C for 6 h. The solution without the silk fiber was kept as a control with the above ascribed conditions. The obtained ZnSk structures were further analyzed.

### 2.3. Catalytic Study

The photocatalytic activity of the obtained ZnSk was investigated by means of naphthalene degradation experiment. Primarily, 80 mg of the ZnSk sample (contains 10 mg of Zn upon on analysis with Multiparameter Photometer) was added to 30 mL naphthalene solution of 43 mg/L in a 100 mL wide mouth glass flask. The investigation was performed at room temperature (34 °C), with continuous stirring (800 rpm) in a rotary shaker incubator. During incubation, the obtained suspensions were irradiated from top under UV lamp (11W), Philips PL-S, Hyderabad, India, a compact UVC source with intensity of 22 lux. Samples for analysis were taken periodically and the decomposition of naphthalene versus irradiation time was monitored by UV–visible spectrophotometer.

For reference measurements, silk fibers devoid of ZnO were also exposed to naphthalene, in the same way as the above stated experimental conditions, and monitored spectrally.

### 2.4. Rose Bengal (RB) Dye Removal

Adsorption experiments were carried out by maintaining a ratio of RB/(ZnSk or silk fibers) as 2.45 mg/g. An orbital incubator shaker (moving at 800 rpmREMI, Mumbai, India) was used for the study, and samples were monitored till 400 min. A pH of ~6.5 and a temperature of ~34 °C were used for investigations devoid of light. The concentration of residual RB was determined using a UV-visible spectrophotometer (UV-3600 Shimadzu, Kyoto, Japan), where the measurements were made at 545 nm, which corresponds to its maximum absorbance. Silk fibers devoid of ZnO are used as control. The change in RB dye amounts were calculated from the concentrations in solutions before and after dye adsorption. The mechanism of adsorption by silk and ZnO deposited fibers were estimated by using the pseudo-first and second-order kinetic models [22]. Additionally, the adsorption behavior of RB dye was estimated using Langmuir adsorption isotherm model. For isotherm studies, ~0.287 g of ZnSk or silk fibers were used in a 50 mL aqueous solution of RB (concentrations 0.005 to 0.03 mM, pH ~ 6.5). All the experiments were done using sterile 100 mL glass flasks, properly covered, and periodic samples were removed for UV-vis spectral analysis.

### 2.5. Antimicrobial Activity of ZnSk Composite

The produced ZnSk composite was tested for bactericidal activity against Gram negative *E. coli*, using the zone of inhibition (ZOI) method. Bacterial cell suspension of 0.1 mL (from 2% *w/v*) was plated uniformly, with the help of spreader on nutrient agar plates. Using a sterile cork borer, 10 mm wells were made on agar plates, and ZnSk composite (of 20 mg) was placed in the well. Here, degummed silk fibers were used as a control and agar plates with samples were then incubated at 37 °C for 24 h. The diameters of the resulting ZOI in mm due to microbial growth were measured using imageJ software on digital images, and the antibacterial activity was determined.

### 2.6. Analysis and Characterization

UV-visible spectra of the samples were observed using Cyberlab UV100, UV-vis spectrophotometer. The scanning electron microscopy (SEM) observations were performed on JEOL-2100F machine (JEOL India Pvt. Ltd, Kolkata, India). X-ray diffraction (XRD) measurements were done by X-ray diffractometer (Philips PW 1830 HT, Amsterdam, Netherlands), with an accelerating voltage of 35 kV, with a current of 30 mA. The photoluminescence (PL) spectra were measured using spectrofluorometer (Hitachi F-7000, Chiyoda, Tokyo, Japan. The Zn content in ZnSk was identified using HANNA HI83399 Multiparameter Photometer.

## 3. Results

### 3.1. Microscopic Analysis

**Figure 1:**
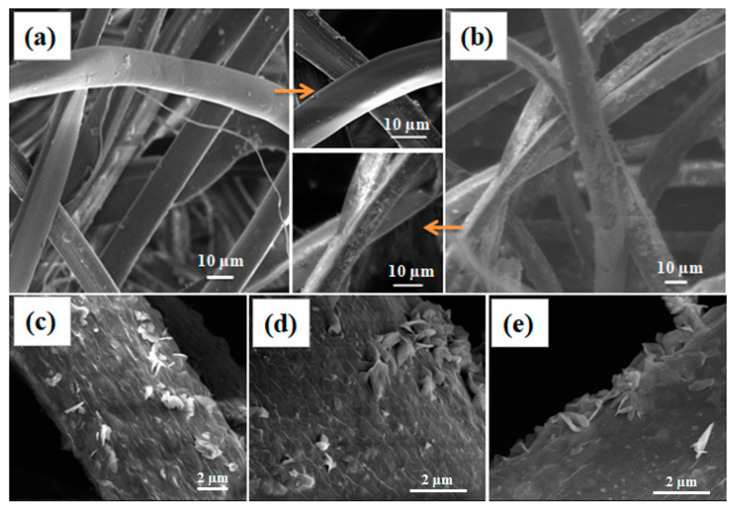
Scanning electron microscopy (SEM) images of (**a**) degummed silk fibers, (**b**) ZnO coated silk fibers. High magnification images from (**b**) are given in (**c**–**e**) where ZnO flake type structures are clearly visible.

Degummed silk fibers with and without the treatment with zinc precursor are shown in Figure 1. While the degummed fibers show smooth morphology (Figure 1a); treated fibers exhibited a layer of particle deposition, due to formation of ZnO nanoparticles throughout the silk fibers surface (Figure 1b). The close magnification of the silk fiber images reveals that the formed ZnO particles are mainly flake type structures with 500–700 nm in length and ~60 nm thicknesses (Figure 1c–e). The top and side views of the fibers show the thickness, orientation, and aggregation of the ZnO flake structures on the surface of silk fibers. At the images (Figure 1d,e), there were few pseudo spherical type structures (160–190 nm), along with ZnO flakes.

In this mode of synthesis, water is the most suitable oxidizing agent, whilst methanol and ethanol facilitate the fast transformation of the precursor mist into vapor form during the heating step, after an addition of the mixtures to the degummed silk fibers. The formation of nanoscale ZnO at 150 °C for 6 h may be described by the following equation:

Zn(CH_3_COO)_2_+ H_2_O + degummed silk fibers → ZnSk composite + 2CH_3_COOH(g)

**Figure 2:**
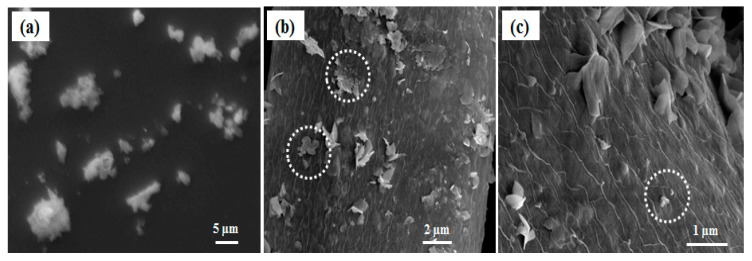
SEM image of (**a**) ZnO particles devoid of silk fibers; (**b**,**c**) shows ZnO nanoflakes on silk fiber (ZnSk) structures and the aggregations of small particles; dotted circular regions in white display aggregations of pseudo smaller spherical particles.

ZnO particle synthesis devoid of silk fibers exhibited no flake type structures and furthermore bigger ZnO particles with a high degree of polydispersity were formed (Figure 2a). This indicates that there is a role of silk fibers in directing the ZnO particles size and shape. Degummed silk fibers are rich in fibroin content, mainly made up of non-polar amino acids, like glycine and alanine, which sum up approximately 76% of their structure [23]. We believe that they secure the growth process of ZnO structures by harboring the initial nuclei formed from the ZnO precursor, and then acting as a growth sites to form ZnO flakes. This hypothesis can be confirmed microscopically in Figure 2b,c, where there exist some aggregations of pseudo smaller spherical particles forming flake type structures on the surface of the silk fibers. We believe that these are the points or places where there is a possibility for growth of ZnO nanoparticles into bigger structures. We assume that the observed ZnO nanoparticles are in between their growth step to form bigger flake type structures.

### 3.2. XRD Analysis

**Figure 3:**
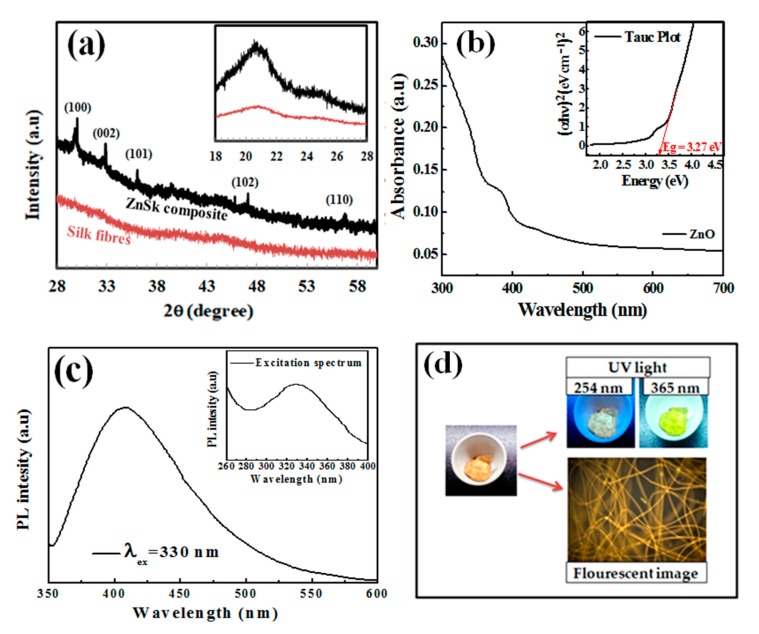
(**a**) X-ray diffraction (XRD) patterns of silk fibers and ZnSk composites. The hexagonal crystal system of ZnO with facets are indicated above the diffraction pattern of ZnSk composites, (**b**) UV-visible spectrum of ZnSk and Tauc plot depiction (inset), (**c**) fluorescence spectra of ZnSk and its excitation spectrum (inset), (**d**) visible emissions from ZnSk composites in a UV box and fluorescence microscopic observation with blue excitation.

The X-ray diffraction spectra of the silk fibers and ZnSk composite are presented in Figure 3a. The silk fibers in Figure 3a exhibit strong peaks with 2θ value around 20.7°, this corresponds to the presence of crystalline domains in silk fibers [24,25]. The crystalline groups of silk include glycine-X repeats covering 94% of silk sequence. Here, X include alanine (65%), serine (23%), and tyrosine (9%) [24]. Coming to the diffraction pattern of ZnO (Figure 3a) deposited on silk fibers, 2θ values observed are to be at 30.26°, 32.91°, 36.08°, 47.08°, 56.79°, and the dashed lines in Figure 3a indicate different facets. The above sample data can be attributed to the hexagonal crystal system of ZnO, and matches near with JCPDS file no: 36-1451, 79-0206 [12,26]. Zn content in ZnSk composite identified to be 125 mg/g. This value is low and might be the reason for low intensity peaks from ZnSk samples.

### 3.3. UV-Vis Spectroscopy Analysis

Figure 3b shows the UV-visible spectrum of dispersed ZnSk in water. Only silk fiber suspension in water was taken as a reference here. The sample shows an absorption maximum in the UV region and the band gap energy was obtained using the observed spectrum. The optical band gap of the synthesized ZnO structures is calculated by using Tauc plot (Figure 3b insert), and was observed to be 3.27 eV. This value is near to a reported study with flake type structures of ZnO [27], and also matches with other studies as well [28,29].

Furthermore, the PL behaviour of ZnSk was observed using a spectrophotometer, and the measurements are shown in Figure 3c. The ZnSk exhibited strong and wide PL signal range from 380 to 500 nm, with the excitation light energy higher than the bandgap energy. The emission and excitation maxima of the ZnO coated silk fiber spectra are observed to be 330 nm and 420 nm, respectively (Figure 3c). The PL signal credited to excitonic PL and is a result of surface oxygen vacancies and defects of the ZnO nanoparticles [30]. The high intensity peak at 420 nm in particular attributed to band edge free excitons of ZnO nanostructures [30]. While the PL behavior from silk fibers is absent, the visible appearance and color changes of the ZnSk are observed using short (254 nm) and long (365 nm) UV irradiation lamp sources using a UV box (Figure 3d). The bright lemon green color emission at 365 nm wavelength exposure is clearly visible in the picture. The fluorescence emission of ZnSk was also visualized through a fluorescence microscope under blue excitation, and the image is shown in Figure 3d.

### 3.4. Degradation Study

Prior to degradation studies, the adsorption effect was monitored by exposing silk fibers and ZnSk composites to naphthalene for 30 min devoid of light. With no or negligible decrease in UV-vis spectrum of naphthalene at 274 nm, it was observed that naphthalene has no adsorption effect towards silk fibers or ZnSk composites.

**Figure 4:**
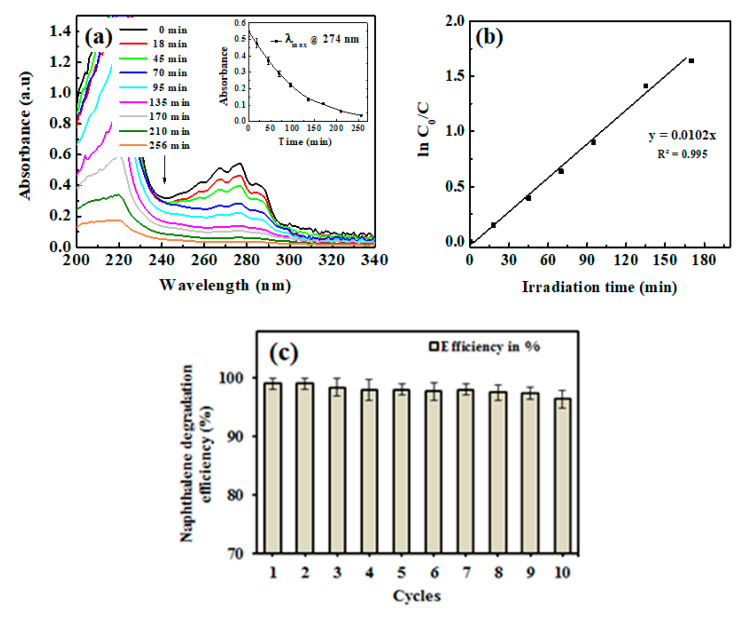
(**a**) UV-visible spectrum of naphthalene during degradation by ZnSk composite, with a plot of kinetic of absorbance change at 274 nm (inset), (**b**) first-order kinetic model fit to the degradation data. (**c**) Reusability of ZnSk composites for naphthalene degradation (80 mg ZnSk exposed to 43 mg/L naphthalene), and error bars represent standard deviation for three measurements.

Next, we studied degradation study, where Figure 4a shows the kinetics of the photocatalytic degradation of naphthalene solution by the ZnSk composites. The time dependent spectral behavior of naphthalene at absorption maximum (λ_max_) of 274 nm is observed, with a decreasing trend in λ_max_ values and up to 93% degradation is observed in 256 min. Silk fibers devoid of ZnO did not displayed any change in λ_max_ value at 274 nm upon UV irradiation, indicating no effect on naphthalene degradation. The prepared ZnSk composites, after repeated use (three times), displayed a decreasing trend of naphthalene absorbance, and the average λ_max_ values at 274 nm with increasing time is shown in Figure 4a inset.

The observed data, in accordance with the ln (C_0_/C) vs irradiation time, fit better with the first-order kinetic model of the rate law (Figure 4b). The rate constant K form the slope of the observed trend line is 0.0102 min^−1^ and the half-life time of degradation is calculated to be 67.94 min. With a higher size ZnO nanoflake type structures, and with low deposition in terms of Zn (125 mg/gm of ZnSk) dose, the degradation behavior of naphthalene is comparable or superior to other reported studies with different nanoparticles (like TiO_2_) and other approaches [31,32]. Furthermore, reports state that the photocatalytic activity of ZnO towards naphthalene is reasonably profound, and the degradation process is OH• radical mediated with quick end products [33,34]. The reaction can be effectively monitored using a simple UV-vis spectroscope, and the steps involved in the reaction are detailed below [33,34].

(1) $$\mathrm{ZnO}+\mathrm{hv}\to e^{-}+h^{+}$$

(2) $$h^{+}+H_{2}O\to H^{+}+{\mathrm{OH}}^{.}$$

(3) $$h^{+}+{\mathrm{OH}}^{-}\to {\mathrm{OH}}^{.}$$

(4) $$e^{-}+O_{2}\to O_{2}^{-}$$

(5) $$2e^{-}+O_{2}+2H^{+}\to H_{2}O_{2}$$

(6) $$e^{-}+H_{2}O_{2}\to {\mathrm{OH}}^{.}+{\mathrm{OH}}^{-}$$

In addition to the above, complementing our study and observations, the enhanced photocatalytic properties of anisotropic ZnO materials are attributed to the high surface area and the nanofeatures [33,34]. These structures result in decrease in chance of recombination for photo-excited electron–hole pairs. The excited electrons are captured by the oxygen vacancies on the surface, and thus restrain the recombination of electrons and holes [33,34]. The holes in the valence band of ZnO attack the surface hydroxyls and yield surface-bound OH• radicals, which participate in photocatalytic reactions effectively. The rate of generation of these OH• radicals is higher in anisotropic ZnO materials and thus promotes more photocatalytic activity. These preliminary results from our study (Figure 4) indicate that ZnSk composites could find promising potential in environmental and wastewater treatment applications.

The synthesized ZnSk structures have huge scope to be used in real time studies, as they are template based and firmly associated with silk fibers after repeated aqueous washings (observed Zn content is 118 mg/gm ZnSk with net loss in terms of Zn being ~4.8% after 12 washings). The application of ZnSk will prevent the leaching out of ZnO, and these structures can be recovered and reused easily, without any trouble. The consistent degradation ability (>97%) of ZnSk composites after 12 cycles (Figure 4c) further ensures its degradation efficiency, and these materials may be used for large scale remediation capability.

### 3.5. RB Removal/Adsorption Study

**Figure 5:**
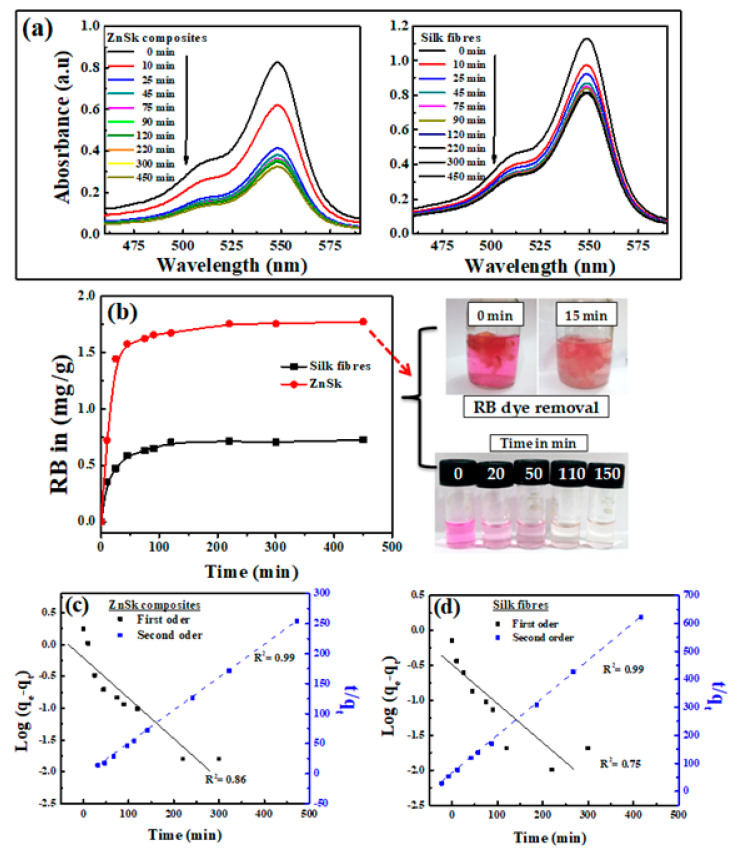
(**a**) UV-visible absorption spectra of Ross Bengal (RB) upon exposure to ZnSk composite and silk fibers. Initial concentration of RB used is 0.017 mM for ZnSk and 0.024 mM for silk fibers (for a 50 mL working volume solution). Weights of adsorbents used are 0.335 g (for ZnSk) and 0.477 g (for silk fiber). (**b**) The absorption threshold is shown in graph with RB color changes in the presence of ZnSk with time as a picture insert. Graphs from (**c**,**d**) correspond to first order and second order models for the adsorption of RB by ZnSk composites and silk fibers.

RB, an anionic dye, was used as a model pollutant to evaluate the adsorption properties of the produced ZnSk composite. As soon as the fibers were exposed to the RB solution, the rose color slowly disappeared, and the typical visible spectrum peak of RB at ~550 nm progressively decreased (Figure 5a). The rate of absorbance decrease was more significant for ZnSk composite compared to silk fibers, and the fibers became colored due to the RB adsorption.

Figure 5b shows the calculated adsorbed amount of RB for an experiment conducted up to 450 min at room temperature (32 °C). Even though the course of adsorption is similar for both of the experiments, the ZnSk composites exhibited markedly greater adsorption ability than the silk fibers devoid of ZnO. The adsorbed amount of RB was ~0.723 mg/g for silk fibers and ~1.770 mg/g for ZnSk. This value indicates the adsorption capacity of ZnSk is about ~2.45 times higher than silk fibers. The saturation time for RB adsorption was found to be ~120 min for both sorbents.

To investigate adsorption controlling mechanism, a pseudo-first order kinetic model was utilized to evaluate the experimental data [22]. The rate of adsorption based on the adsorption capacity in its linear form through pseudo-first order kinetic model is expressed as follows:

(7) $$\log\left(q_{e}-q_{t}\right)=\log q_{e}-\frac{k_{1}}{2.303}t$$

Furthermore, the pseudo-second order model is presented as follows:

(8) $$\frac{t}{q_{t}}=\frac{1}{k_{2}q_{e}^{2}}+\frac{1}{q_{e}}t$$

$$h=k_{2}q_{e}^{2}$$

where q_e_ (mg/g) is adsorption capacity, q_t_ is adsorption at time t and k_1_ (min^−1^), k_2_ (g mg^−1^ min^−1^) are pseudo-first and pseudo-second order rate constants, and h (mg g^−1^ min^−1^) is the initial adsorption rate, corresponding to the second-order kinetic model.

The adsorption kinetics was estimated using Equations (1) and (2) to get information on the adsorption behavior with respect to time. Batch adsorption data using the pseudo-first and second-order kinetic models are presented in Figure 5c,d for ZnSk and silk fibers individually. Correlation coefficient (R^2^) values for the pseudo-first order kinetics are 0.86 and 0.75 for ZnSk and silk fibers respectively, while R^2^ values for the second-order model are 0.99 and 0.99 for the same. A better correlation between the modelled and experimental data was observed using the pseudo-second order kinetics model. This favors chemisorption, and this behavior is the rate controlling factor for the adsorption of RB onto silk fibers and ZnSk composite used. The observed performance might be true, as the degummed silk fibers have the ability to fix anionic dyes through ionic bonding. Here, positive –NH_2_ group of the fiber will bind to acid group of dye [35].

Furthermore, for examination of the adsorption behavior with different RB dye concentration, a quantitative Langmuir adsorption isotherm model was utilized to evaluate the experimental data. Langmuir equation in its linear form is usually expressed as:

(9) $$\frac{C_{e}}{Q_{e}}=\frac{1}{Q_{m}K}+\frac{C_{e}}{Q_{m}}$$

where, Q_e_ is equilibrium dye concentration on adsorbent (mg g^−1^), Q_m_ is monolayer capacity of the adsorbent (mg g^−1^), K is adsorption constant (L mg^−1^), and C_e_ is equilibrium dye concentration in solution (mg L^−1^).

According to the above equation, a plot of C_e_/Q_e_ versus C_e_ should be a straight line with a slope 1/Q_m_ and intercept 1/Q_m_K.

**Figure 6:**
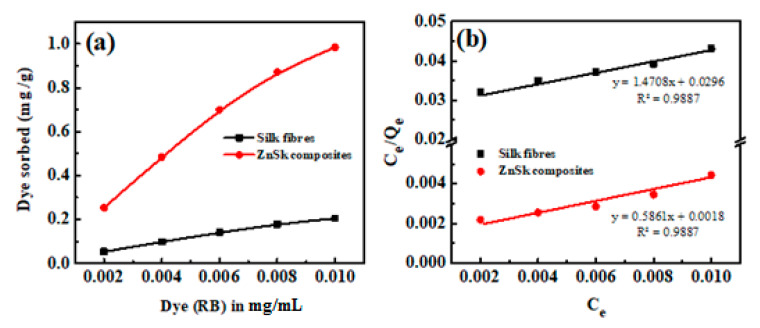

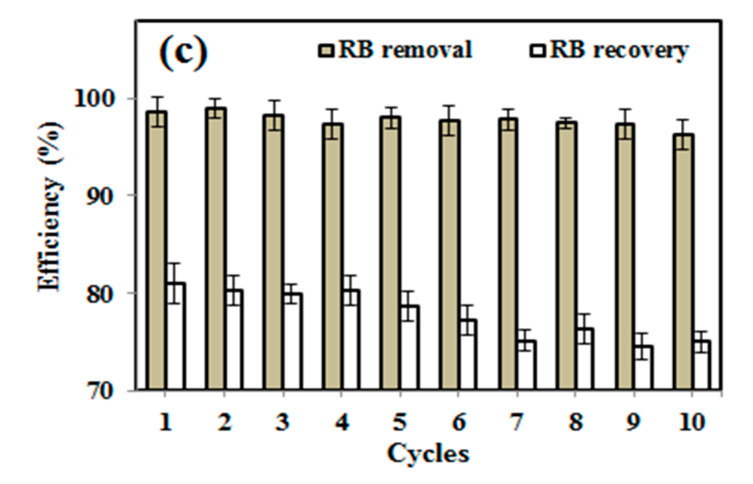
Using silk fibers and ZnSk composites, (**a**) RB adsorption vs. concentration and (**b**) linear plots showing the Langmuir adsorption isotherm parameters. (**c**) RB removal and recovery studies using ZnSk composites. Error bars are calculated standard deviation for three measurements.

The equilibrium adsorption isotherm is of primary importance in the design of adsorption process. The Langmuir adsorption model is applied to the experimental data obtained from various RB concentrations and its adsorption (Figure 6a). A plot of C_e_/Q_e_ vs. C_e_ is given in Figure 6b, and the calculated adsorption constant (K) values are 0.325 and 0.0497 (L mg^−1^), and Q_m_ values are found to be ~1.706 and 0.679 mg g^−1^ for ZnSk and silk fibers, respectively. The observed Q_m_ values are promising and better than reported values using bottom ash type adsorbents [36] and Fe(III)–montmorillonite clay material [37]. The maximum RB adsorption achieved is ~0.04 mM g^−1^ in the former case, and it is 0.868 mg/g in the later reported study. The reusability of ZnSk composites are tested by exposing them to 0.006 mM RB with a ZnSk dose of 0.287 g in 50 mL solution (i.e., RD/ZnSk is 1 mg/g) for 12 cycles, and results are shown in Figure 6c. Values in the figure indicate a decent performance with ~97% RB removal ability after every wash cycle, whereas RB recovery efficiency was bit reduced after six cycles. This performance may be due to experimental error and Zn loss due to washing steps (as stated in Section 3.4). Overall, ZnSk composites are still better after 12 cycles of dye removal, and have the potential to be used in real time settings.

### 3.6. Antimicrobial Activity of ZnO Composites

The most commonly used nanoparticles for water and wastewater treatment are Ag, TiO_2_, and ZnO [38]. These particles with various sizes and shapes have huge scope as a new class of antimicrobial agents in the water disinfection and treatment fields. The applications of Ag and TiO_2_ in real time settings are limited, due to cost and health effects. For instance, TiO_2_ nanostructures could cause genetic mutations in human beings when applied in higher quantities [39]. In this scenario, being safe, stable, and non-toxic, nano ZnO is the principal choice, and has attracted interest as an antimicrobial agent, along with its exotic applications, which range from photocatalysis and sensors to biomedical applications [38,39,40].

**Figure 7:**
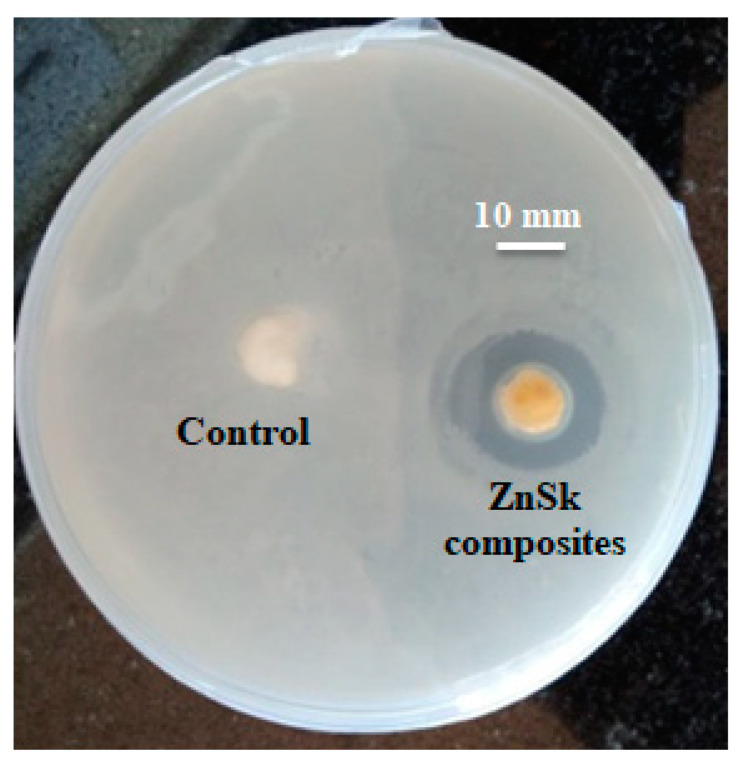
Zone of inhibition test showing antimicrobial effect of ZnSK composite. Degummed silk fibers are used as a control.

The antimicrobial activity of as synthesized ZnSk composite was tested on *E. coli*, and the results are presented in Figure 7. ZOI results showed significant antimicrobial property by ZnSk composite, with the observed inhibition zone of 1.2 ± 0.1 mm, and silk fibers used as control are insensitive to tested *E. coli*. ZOI at ambient room temperature and at normal day light condition suggests that ZnSk composite have promising antimicrobial action. There are various mechanisms of antimicrobial activity of nanoscale ZnO are reported by research groups and key factors of toxicity include size, shape, reactive oxygen species (ROS) production, hydrogen peroxide generation, attachment to bacterial cell surfaces, etc. [1,38,39,40]. Coming to ZnSk composite in this study, ZnO nanoflakes are deposited steadily onto the silk fibers, and the most possible mechanism of toxicity may be due to generation of ROS. The release of ROS by ZnO nanoparticles will result in cause of oxidative stress, DNA damage, decomposition of the cell wall, membrane leakage of reducing sugars, proteins, etc. [38,40], which are identified reportedly.

## 4. Conclusions

In this study, biopolymer, silk fiber was used as a template to synthesize ZnO nanoflakes with zinc acetate as a starting material. This biomimetic approach uses a simple hydrothermal process (with reaction temperature of 150 °C for 6 h) for the growth and formation of ZnO- silk fiber (ZnSk) composites. The ZnSk composite structure was visualized through the electron microscopy, and the results reveal the size of and morphology of the nanoflakes (~500–700 nm in length; ~60 nm thicknesses). The deposition of ZnO was confirmed by XRD, SEM, UV, and fluorescence studies. The efficiency of ZnSk composites in naphthalene degradation is demonstrated. The preliminary measurements of the photocatalytic property of ZnSk composites demonstrate more than 97% degradation ability, along with the reusability of the photocatalyst. Furthermore, taking the advantage of ZnSk composite’s anionic dye adsorption capacity, we used RB for adsorption studies. The maximum RB adsorption capacity was found to be ~1.706 mg g^−1^ of ZnSk composite material, and the experimental data fitted better to the Langmuir model. In addition to the above, with a potent antimicrobial activity against the tested *E. coli* at ambient conditions, results suggest that the multifunctional use of the produced ZnSk composites could have huge scope to be used in environmental, industrial, and wastewater treatment applications.
